# Non-canonical role of the sympathetic nervous system in the day–night rhythm in heart rate

**DOI:** 10.1098/rstb.2022.0179

**Published:** 2023-06-19

**Authors:** Cali Anderson, Gabriella Forte, Wei Hu, Henggui Zhang, Mark R. Boyett, Alicia D'Souza

**Affiliations:** ^1^ Division of Cardiovascular Sciences, University of Manchester, 46 Grafton Street, Manchester M13 9NT, UK; ^2^ Department of Physics and Astronomy, University of Manchester, Schuster Building, Brunswick Street, Manchester M13 9PL, UK; ^3^ Faculty of Life Sciences, University of Bradford, Richmond Road, Bradford BD7 1DP, UK

**Keywords:** heart rate, sinus node, pacemaking, circadian rhythm, sympathetic nervous system

## Abstract

Although, for many decades, the day–night rhythm in resting heart rate has been attributed to the parasympathetic branch of the autonomic nervous system (high vagal tone during sleep), recently we have shown that there is a circadian clock in the cardiac pacemaker, the sinus node, and the day–night rhythm in heart rate involves an intrinsic rhythmic transcriptional remodelling of pacemaker ion channels, particularly *Hcn4*. We have now investigated the role of the sympathetic branch of the autonomic nervous system in this and shown it to have a non-canonical role. In mice, sustained long-term block of cardiac β-adrenergic receptors by propranolol administered in the drinking water abolished the day–night rhythm in pacemaking in the isolated sinus node. Concomitant with this, there was a loss of the normal day–night rhythm in many pacemaker ion channel transcripts. However, there was little or no change in the local circadian clock, indicating that the well-known day–night rhythm in sympathetic nerve activity is directly involved in pacemaker ion channel transcription. The day–night rhythm in pacemaking helps explain the occurrence of clinically significant bradyarrhythmias during sleep, and this study improves our understanding of this pathology.

This article is part of the theme issue ‘The heartbeat: its molecular basis and physiological mechanisms’.

## 1. Introduction

There is a day–night rhythm in the resting heart rate, which is faster during the awake period (day for the human but night for the nocturnal mouse) in preparation for increased levels of physical activity at this time [1]. Since 1929 this has been attributed to the autonomic nervous system [2,3]. The autonomic nervous system of course is known to regulate heart rate via its actions on the pacemaker of the heart, the sinus node—adrenaline, noradrenaline and acetylcholine released from the autonomic nerves innervating the sinus node regulate single-ion channel conductances and therefore heart rate *over a timescale of seconds* (via G protein, cAMP and phosphorylation) [4–7]. It has long been supposed that there is a day–night rhythm in the activity of the autonomic nerves innervating the sinus node—attention has focused on a potential increase in vagal nerve activity during the sleep period [1]. An increase in heart rate variability has been cited as evidence of this (e.g. [8]), but we have shown that heart rate variability cannot be used as a measure of vagal activity [9]. Furthermore, two studies have shown there is no day–night variation in vagal activity [10,11]. However, there is evidence of a day–night rhythm in sympathetic nerve activity [10,12], as well as a day–night rhythm in plasma catecholamines released from the adrenal medulla [13–15] and in the catecholamine content of the heart [16]; all are greater during the awake period. Although this potentially could explain the higher resting heart rate during the awake period (via changes in single-ion channel conductances), short-term pharmacological autonomic blockade does not block the day–night rhythm in the resting heart rate [1]. Our recent work has turned attention away from the autonomic nervous system to the sinus node itself. We have shown (i) there is a local circadian clock in the sinus node, (ii) 44% of the transcriptome of the sinus node shows a significant day–night rhythm, including transcripts underlying pacemaking such as *Hcn4*, (iii) a day–night rhythm in pacemaker activity is seen in the isolated (therefore denervated) sinus node, (iv) there is a day–night rhythm in the funny current, *I*_f_, for which *Hcn4* is responsible, and (v) block of *I*_f_ *in vitro* and *in vivo* diminishes or blocks the day–night rhythm in heart rate [17,18]. Our work therefore suggests that the day–night rhythm in the resting heart rate in part at least is intrinsic to the heart itself. However, questions remain. In contrast with short-term pharmacological autonomic blockade, long-term pharmacological sympathetic nervous system blockade reduces or abolishes the day–night rhythm in the resting heart rate [1,19–21]. This raises the possibility of a non-canonical role (i.e. one other than the regulation of single-ion channel conductances) for the sympathetic nervous system in the day–night rhythm in the resting heart rate, and the aim of this study was to investigate this possibility.

## 2. Results

### (a) Effect of sustained β-adrenergic receptor blockade *in vivo*

**Figure 1.**
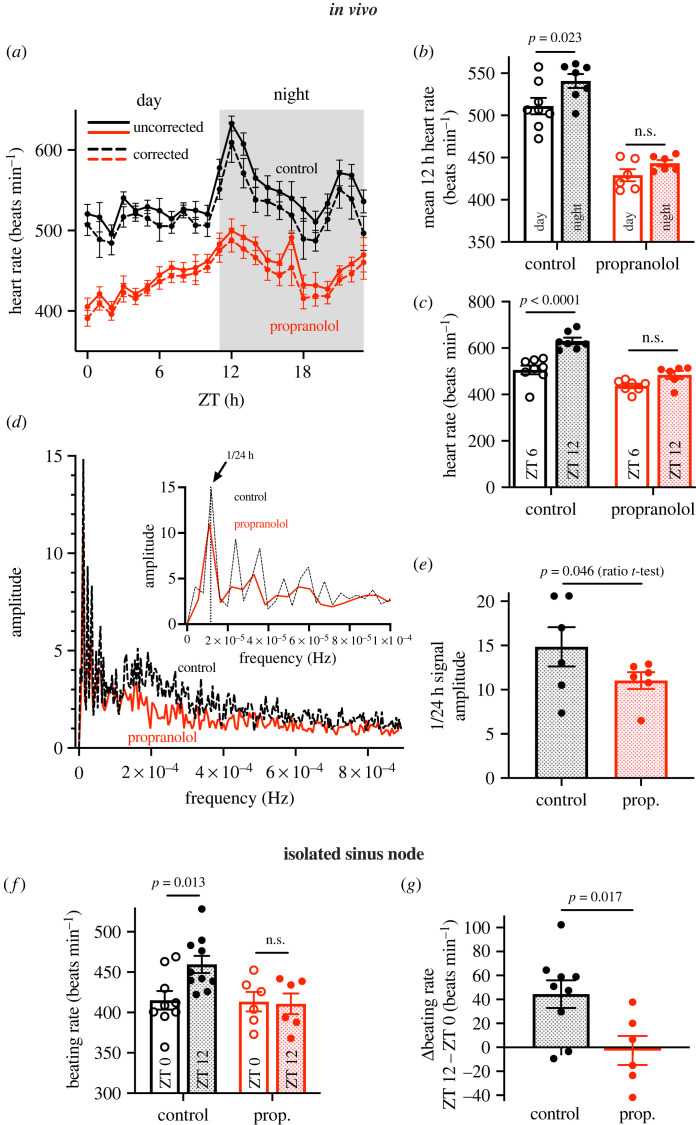
Effect of sustained β-adrenergic receptor blockade on the day–night rhythm in heart rate. (*a*) Mean hourly uncorrected (solid lines) and activity-corrected (dashed lines) heart rate measured by telemetry over a 24 h cycle in control and propranolol-treated mice (*n* = 7 or 8 per group). (*b*) Activity-corrected heart rates measured by telemetry during the day (12 h light period) and night (12 h dark period) in control and propranolol-treated mice (*n* = 6–8 per group). (*c*) Activity-corrected heart rates measured over 1 h by telemetry at ZT 6 and ZT 12 in control and propranolol-treated mice (*n* = 6–8 per group). (*d*) Mean fast Fourier transform of the heart rate (not activity-corrected) from control and propranolol-treated mice over at least 48 h (*n* = 6 per group). Inset shows details of the transform at the lowest frequencies (dotted line shows a frequency of 1 per 24 h). (*e*) Amplitude of the 1 per 24 h component of the fast Fourier transform in control and propranolol-treated mice (*n* = 6 per group). (*f*) *Ex vivo* beating rate measured by extracellular potential recording in right atrial preparations isolated at ZT 0 and ZT 12 from control (*n* = 9 or 10 per time point) and propranolol-treated (*n* = 6 per time point) mice. (*g*) Difference in the beating rate between ZT 12 and ZT 0 (same data as in (*f*)). prop., propanolol. Differences were tested for statistical significance using a mixed-effects model with Šídák's multiple comparisons test (*b*,*c*,*f*) or Student's *t-*test (*e*,*g*). *p*-values shown; n.s., not significant. Data are shown as means (*d*), means ± s.e.m. (*a*), and means ± s.e.m. and individual biological replicates (*b*,*c*,*e*–*g*). (Online version in colour.)

Adult male mice were subjected to sustained β-adrenergic receptor blockade by propranolol dosing in the drinking water (3.5 mg day^−1^) for a total of 15–21 days (electronic supplementary material, figure S1*a*); this and similar protocols have been widely used (e.g. [21]). As compared with control mice (of same sex and age, but given standard drinking water), propranolol treatment had no effect on body weight; it also had no effect on the PR interval, QRS duration or corrected QT interval measured from the electrocardiogram (ECG) at zeitgeber time (ZT) 6 in anaesthetized mice (electronic supplementary material, figure S1*b*,*d*–*f*). However, sustained β-adrenergic receptor blockade led to a reduction in the heart rate in freely moving conscious mice (measured using telemetry; figure 1*a* and electronic supplementary material, figure S1*g*). A similar reduction in the heart rate was also observed in conscious but constrained mice (measured using the ECGenie; electronic supplementary material, figure S1*h*) as well as anaesthetized mice (measured using conventional electrodes; electronic supplementary material, figure S1*c*). The reduction in heart rate is expected and is the result of the block of sympathetic neurotransmission to the sinus node. To test the efficacy of the blockade, mice were given an intraperitoneal injection of the non-selective β-adrenoceptor agonist isoprenaline (2 mg kg^−1^) under anaesthetic at ZT 6; this showed that the blockade was greater than 70% (electronic supplementary material, results and figure S2). In mice, sustained β-adrenergic receptor blockade has been reported to reduce locomotor activity during the night (when mice are active) [22], and a similar decrease in locomotor activity (measured using telemetry) was observed in the present study (electronic supplementary material, figure S3*a*–*c*). Sympathetic activity to the heart is expected to follow locomotor activity levels, and this could influence the data from this study. Figure 1*a* shows the mean heart rate *in vivo* measured using telemetry over 24 h only during 1 min periods of inactivity (dashed lines) as well as at all times (solid lines); correction of heart rate for locomotor activity in this manner only had a modest effect on the data.

### (b) Sustained β-adrenergic receptor blockade reduces the day–night rhythm in heart rate *in vivo* and *in vitro*

Figure 1*a* shows that in control mice, as in other studies (e.g. [17]), there was a day–night rhythm in heart rate, which was higher during the awake period (night). As an aside, figure 1*a* also shows that the day–night rhythm in heart rate was similar regardless of whether the heart rate was corrected for locomotor activity, and this suggests that the day–night rhythm in heart rate was not contingent on fluctuations in locomotor activity, in line with our previous study [17]. Not only was the heart rate reduced after sustained β-adrenergic receptor blockade, the day–night rhythm in heart rate was diminished (figure 1*a*). Figure 1*b* shows the mean 12 h heart rate during the day and at night. This shows that the heart rate was significantly higher at night in control mice but the day–night difference in heart rate was no longer significant after sustained β-adrenergic receptor blockade. The heart rate was lowest at ZT 0 and highest at ZT 12, but the difference in heart rate between these two times points was not significantly affected by sustained β-adrenergic receptor blockade (data not shown). However, the difference in heart rate between ZT 6 and ZT 12 was significantly smaller (*p* = 0.031) following sustained β-adrenergic receptor blockade (figure 1*c*). The pattern of the day–night rhythm in heart rate was altered after sustained β-adrenergic receptor blockade, and this was investigated using a fast Fourier transform of the data (figure 1*d*). The transform converts a waveform (the day–night rhythm in this instance) into its different frequency components—the transform is shown in the main panel of figure 1*d*, and the lowest frequency components are shown in the inset in figure 1*d*. As expected, the main component had a frequency of 1 per 24 h (the dotted line in the inset in figure 1*d* corresponds to a frequency of 1 per 24 h), and the amplitude of this component was significantly reduced following sustained β-adrenergic receptor blockade (figure 1*e*). Figure 1*d* shows that the amplitude of higher-frequency components was also reduced following sustained β-adrenergic receptor blockade. In conclusion, the day–night rhythm in heart rate *in vivo* was significantly dampened following sustained β-adrenergic receptor blockade.

To study the effect of sustained β-adrenergic receptor blockade on the intrinsic pacemaker activity of the sinus node, extracellular potentials were recorded from the sinus node isolated at ZT 0 and ZT 12 from control mice and mice following sustained β-adrenergic receptor blockade. The beating rate of the isolated sinus node was significantly higher at ZT 12 as compared with ZT 0 in control mice, but in mice following sustained β-adrenergic receptor blockade this difference was abolished (figure 1*f,g*). Interestingly, this was the result of a reduction of the beating rate at ZT 12; there was no change in the beating rate at ZT 0 (figure 1*f*). These effects are consistent with data from mice *in vivo* (figure 1*a*–*g*). In conclusion, the day–night rhythm in intrinsic sinus node pacemaking was abolished following sustained β-adrenergic receptor blockade.

### (c) Local circadian clock transcripts in the sinus node retain their rhythmicity following sustained β-adrenergic receptor blockade

**Figure 2.**
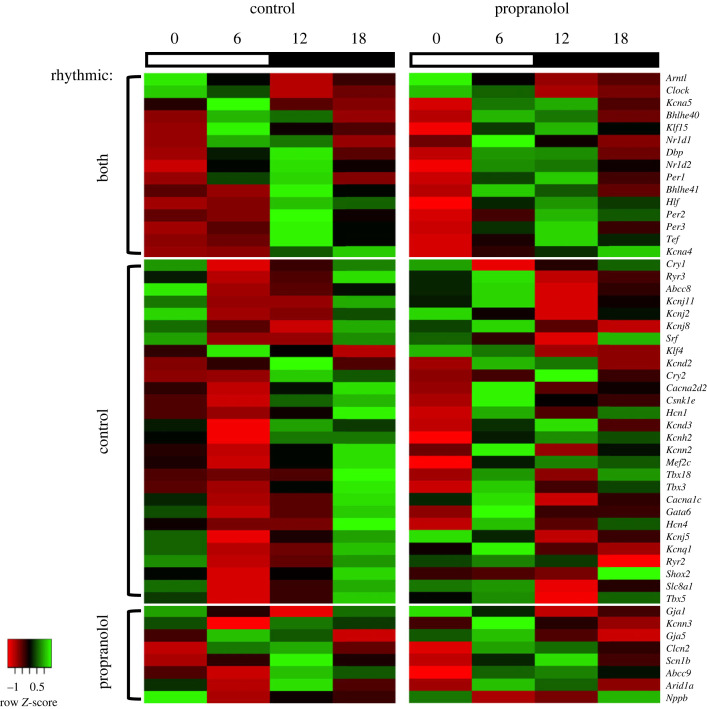
Heat maps of transcripts that retained (both), lost (control) or gained (propranolol) rhythmicity in the sinus node after sustained β-adrenergic receptor blockade. Mean expression of transcripts showing a day–night rhythm (confirmed by JTK_CYCLE) in both control and propranolol-treated mice or just control or propranolol-treated mice is shown at ZT 0, 6, 12 and 18 (*n* = 4 or 5 per measurement). Expression is colour-coded according to the *Z*-score (deviation from the mean measured in terms of s.d.) and goes from red (low expression) to green (high expression). Transcripts in each section are ordered by the time of peak expression (LAG value from JTK_CYCLE). Heatmaps constructed using the online tool heatmapper.ca without clustering. (Online version in colour.)

We have previously shown that there is a local circadian clock in the sinus node and in addition there is a day–night rhythm in pacemaker ion channel transcripts (e.g. in *Hcn4*) and this is responsible for or plays a role in the day–night rhythm in heart rate [17,18]. Previously we suggested that the local clock is driving the day–night rhythm in pacemaker transcripts [17,18]. TaqMan quantitative polymerase chain reaction (qPCR) array cards were therefore used to look for changes in transcripts that could be responsible for the attenuation of the day–night rhythm in heart rate following sustained β-adrenergic receptor blockade. The expression of 90 transcripts involved in circadian clock function and sinus node pacemaking was measured at four time points across the 24 h cycle (ZT 0, 6, 12 and 18) in control mice and mice following sustained β-adrenergic receptor blockade. Statistical significance of 24 h oscillations was tested using JTK_CYCLE software [23]. In total, 51 of the 90 transcripts showed statistically significant day–night rhythms (figure 2); of these, 17 transcripts were rhythmic in both groups of mice. A further 26 transcripts were rhythmic in the control mice only, and eight genes were uniquely rhythmic in the mice following sustained β-adrenergic receptor blockade.

**Figure 3.**
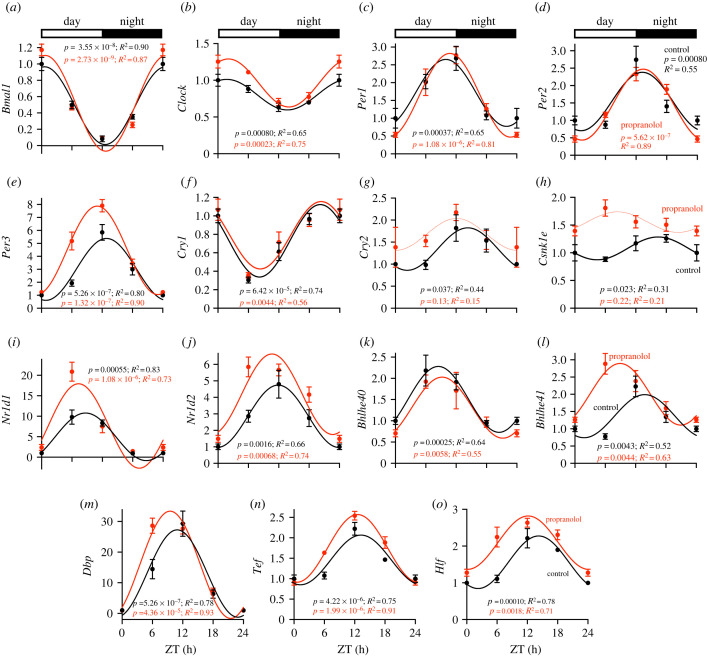
Expression of sinus node clock transcripts and transcripts for clock-controlled transcription factors across the 24 h cycle in control and propranolol-treated mice. In this and similar figures, transcript expression is shown at ZT 0, 6, 12 and 18 in control and propranolol-treated mice (*n* = 4 at ZT 0 for the control mice; otherwise *n* = 5 for each time point and group); expression at ZT 0 is replotted at ZT 24 as a visual aid. Data (shown as means ± s.e.m.) are normalized to the ZT 0 mean in control mice. In the case of transcripts showing a significant day–night rhythm (as determined by JTK_CYCLE), the data are fitted with a sine wave shown as a solid line. In the case of transcripts not showing a significant day–night rhythm, the data are fitted with a sine wave to guide the eye, but it is shown as a dotted line. The adjusted *p*-value (from JTK_CYCLE) and *R*^2^ value (measure of the goodness of fit of the sine wave to the mean data; from GraphPad Prism version 9) for the control and propranolol-treated conditions are shown. (Online version in colour.)

Surprisingly, most of the transcripts that retained rhythmicity in the mice following sustained β-adrenergic receptor blockade were circadian clock transcripts or transcripts for established clock-controlled transcription factors. Rhythmicity was retained in the core circadian clock transcripts (*Bmal1*, *Clock*, *Per1*, *Per2*, *Per3* and *Cry1*; figure 3*a*–*f*), although *Clock* (figure 3*b*) and *Per3* (figure 3*e*) displayed increased amplitude. However, rhythmicity was lost in *Cry2* (figure 3*g*). Rhythmicity was also lost in *Csnk1e* (casein kinase 1ε; figure 3*h*), which phosphorylates PER proteins to mark them for degradation. Both *Nr1d1* (Rev-Erbα; figure 3*i*) and *Nr1d2* (Rev-Erbβ; figure 3*j*) retained rhythmicity, but oscillated with increased amplitude. While no change was observed in *Bhlhe40* (DEC1; figure 3*k*), a phase advance and reduction in amplitude was observed for *Bhlhe41* (DEC2; figure 3*l*). The clock-controlled output transcription factor transcripts, *Dbp*, *Tef* and *Hlf*, also displayed an increased amplitude of oscillation (figure 3*m*–*o*). A 24 h oscillation in circadian clock transcripts in the left ventricle also persisted following sustained β-adrenergic receptor blockade (with the exception of *Csnk1e*; electronic supplementary material, figure S4).

### (d) Abolition of the day–night rhythm in pacemaker ion channel transcripts in the sinus node following sustained β-adrenergic receptor blockade

**Figure 4.**
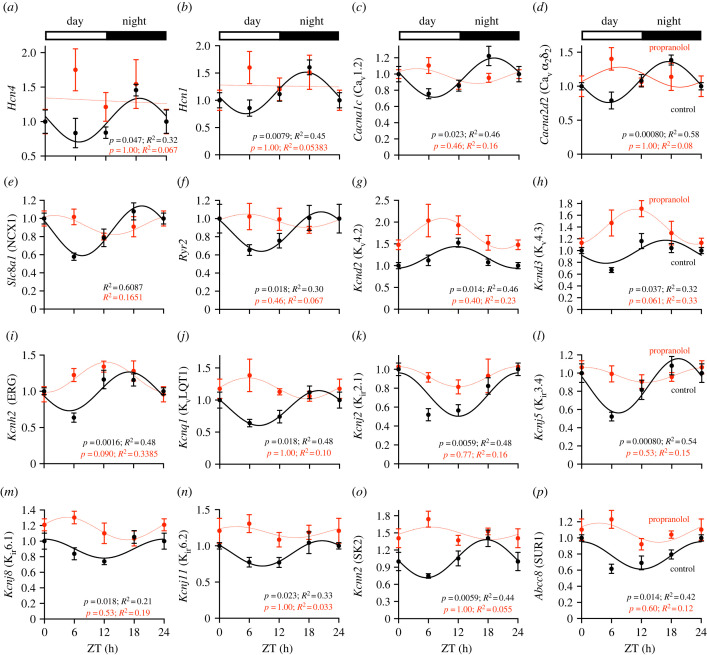
Expression of sinus node ion channel and Ca^2+^-handling transcripts across the 24 h cycle in control and propranolol-treated mice. In all cases, the transcripts show a significant day–night rhythm in control but not propranolol-treated mice. (Online version in colour.)

A loss of the day–night rhythm in many important pacemaker ion channel transcripts was observed in the sinus node following sustained β-adrenergic receptor blockade. For example, there was a loss of a significant day–night rhythm in the case of *Hcn4* (figure 4*a*) and the less abundant isoform, *Hcn1* (figure 4*b*); we have previously demonstrated that *Hcn4* plays an important role in the day–night rhythm in heart rate [17], and therefore the loss of its rhythmicity may play an important role in the reduction in the day–night rhythm in heart rate following sustained β-adrenergic receptor blockade. Rhythmicity was also lost in transcripts for the voltage-gated Ca^2+^ channel, *Cacna1c* (Ca_v_1.2; figure 4*c*), and the Ca^2+^ channel accessory subunit, *Cacna2d2* (Ca_v_α2δ2; figure 4*d*), which is known to be upregulated in the sinus node compared with the working myocardium [24,25]. Following sustained β-adrenergic receptor blockade, rhythmicity in the Na^+^–Ca^2+^ exchanger *Slc8a1* (NCX1; figure 4*e*) and the ryanodine receptor *Ryr2* (figure 4*f*) was lost. The *Ryr3* isoform retained rhythmicity, but oscillated with a dampened amplitude and an altered phase (electronic supplementary material, figure S5*a*). Several K^+^ channel transcripts were altered following sustained β-adrenergic receptor blockade. Transcripts for the transient outward K^+^ channels *Kcnd2* (K_v_4.2; figure 4*g*) and *Kcnd3* (K_v_4.3; figure 4*h*) lost rhythmicity, whereas transcript for the transient outward K^+^ channel *Kcna4* (K_v_1.4; electronic supplementary material, figure S5*b*) oscillated at increased amplitude with higher expression. Transcript for the delayed rectifier K^+^ channel *Kcna5* (K_v_1.5; electronic supplementary material, figure S5*c*) displayed a phase alteration, whereas other delayed rectifier K^+^ channel transcripts, *Kcnh2* (ERG; figure 4*i*) and *Kcnq1* (KvLQT1; figure 4*j*), lost rhythmicity. Transcripts for the inward rectifier K^+^ channels *Kncj2, Kcnj5, Kcnj8* and *Kcnj11* (K_ir_2.1, K_ir_3.4, K_ir_6.1 and K_ir_6.2; figure 4*k*–*n*), as well as miscellaneous K^+^ channels (*Kcnn2*/SK2 and *Abcc8*/SUR1; figure 4*o*,*p*) also lost rhythmicity. Interestingly, several ion channel transcripts (*Scn1b*, *Kcnn3*/SK3, *Clcn2* and *Abcc9*/SUR2; electronic supplementary material, figure S5*d*–*g*) and two gap junction transcripts (*Gja1*/Cx43 and *Gja5*/Cx40) gained rhythmicity following sustained β-adrenergic receptor blockade (electronic supplementary material, figure S5*h*,*i*).

In summary, following sustained β-adrenergic receptor blockade, there was a loss of the day–night rhythm in important pacemaker ion channel transcripts in the sinus node, such as *Hcn4,* and this can explain the loss or reduction of the day–night rhythm in heart rate following sustained β-adrenergic receptor blockade. However, key circadian clock transcripts in the sinus node retained their rhythmicity following sustained β-adrenergic receptor blockade and this has important implications for our understanding of the mechanism underlying the day–night rhythm in the pacemaker transcripts (see Discussion).

Following sustained β-adrenergic receptor blockade, we also observed alterations in the rhythmic expression of several transcripts in the left ventricle that are known to underpin excitation–contraction coupling, including *Ryr2*, *Slc8a1* and *Pln* (ryanodine receptor, Na^+^–Ca^2+^ exchanger and phospholamban; electronic supplementary material, figure S6*a*–*c*). Interestingly, in the left ventricle, there was a marked change in *Kcnd2* (K_v_4.2; electronic supplementary material, figure S6*f*), which has previously been shown to be altered in the ventricle following sustained double autonomic blockade [20], and in *Kcnh2* (ERG; electronic supplementary material, figure S6*g*), which is known to be regulated by the local cardiac circadian clock [26].

### (e) Sinus node transcription factors lose rhythmicity following sustained β-adrenergic receptor blockade

**Figure 5.**
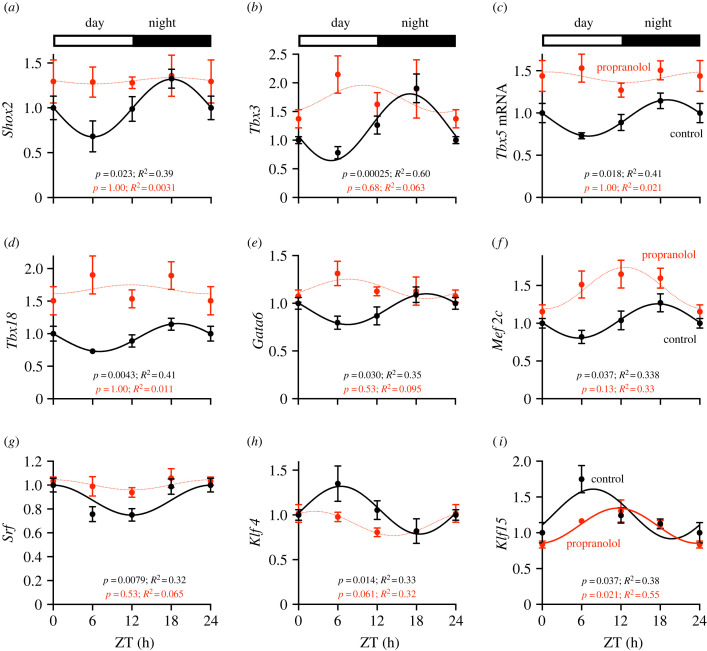
Expression of transcripts for sinus node-specific and ubiquitous transcription factors across the 24 h cycle in control and propranolol-treated mice. (Online version in colour.)

Several transcription factor transcripts known to be important for regulating the development and function of the sinus node show a day–night rhythm [18]. Following sustained β-adrenergic receptor blockade, *Shox2* (short stature homeobox 2; figure 5*a*) and *Tbx3*, *Tbx5* and *Tbx18* (T-box transcription factor transcripts; figure 5*b*–*d*)—all of which were enriched in the sinus node compared with the left ventricle (data not shown)—lost their rhythmicity. Two additional cardiac-enriched transcription factor transcripts also displayed perturbed rhythms: *Gata6* (GATA-binding protein 6; figure 5*e*) and *Mef2c* (myocyte enhancer factor 2c; figure 5*f*). Three ubiquitously expressed transcription factor transcripts, which are known to play a role in maintaining the cardiac gene programme, were also altered following sustained β-adrenergic receptor blockade: *Srf* (serum response factor; figure 5*g*) and *Klf4* (Kruppel-like factor 4; figure 5*h*) lost rhythmicity, while *Klf15* (Kruppel-like factor 15; reported to regulate 75% of the oscillatory transcripts in the heart [27]) showed a phase delay (figure 5*i*). It is possible that the loss of the day–night rhythm in these transcription factors plays a role in the loss of the day–night rhythm in the pacemaker transcripts. *Mef2c* and *Klf15* oscillations were also altered in the left ventricle (electronic supplementary material, figure S6*p*,*q*).

### (f) *Hcn4* function is altered following sustained β-adrenergic receptor blockade

**Figure 6.**
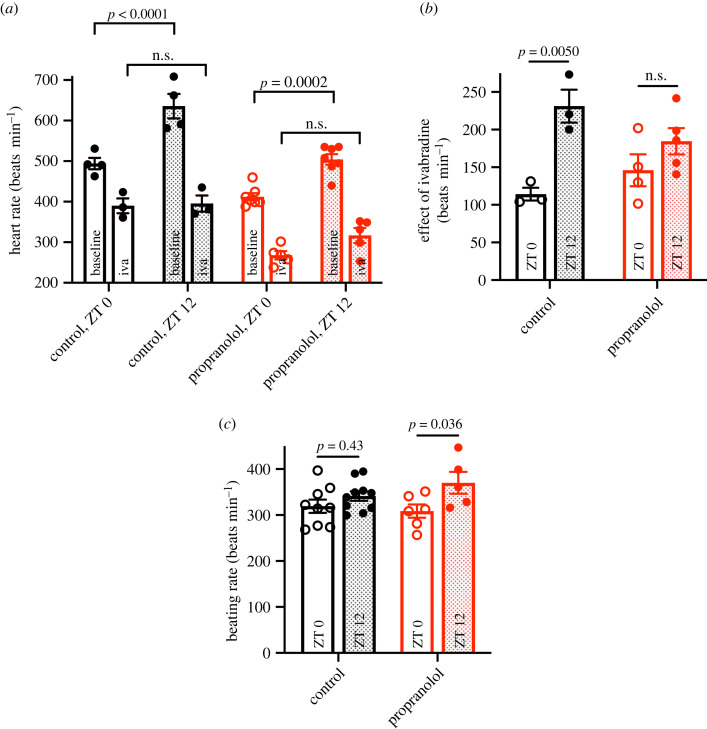
Evidence that the suppression of the day–night rhythm in heart rate in propranolol-treated mice is the result of the loss of the day–night rhythm in HCN pacemaker ion channels. (*a*) *In vivo* heart rate (not activity-corrected) measured by ECG telemetry after intraperitoneal injection of the HCN blocker ivabradine (6 mg kg^−1^) at ZT 0 and ZT 12 in control (*n* = 3 or 4 per time point) and propranolol-treated (*n* = 5 or 7 per time point) mice. Baseline average heart rate at ZT 0 and ZT 12 was calculated for 1 h over several days. In the case of ivabradine (iva), heart rate was calculated as the mean over 30 min starting 10 min after the injection at ZT 0 and ZT 12. Time-dependent differences were tested for statistical significance using a two-way ANOVA with Tukey's multiple comparisons test. (*b*) Effect of ivabradine on the heart rate at ZT 0 and ZT 12 in control and propranolol-treated mice. From the same data as (*a*). (*c*) Beating rate of the sinus node isolated from control and propranolol-treated mice at ZT 0 and ZT12 after block of the funny current, *I*_f_, by 2 mM Cs^+^ (*n* = 5–10 per time point and group). Differences were tested for statistical significance using a two-way ANOVA with Šídák's or Tukey's multiple comparisons test. Means ± s.e.m. and individual biological replicates shown. (Online version in colour.)

Figure 6*a* shows the heart rate *in vivo* at ZT 0 and ZT 12 in control mice and the mice following sustained β-adrenergic receptor blockade under baseline conditions and after the injection of ivabradine to block the funny current, *I*_f_ (for which *Hcn4* and other *Hcn* transcripts are responsible). Figure 6*a* shows that the day–night difference in heart rate was abolished by block of *I*_f_ by ivabradine; the day–night difference in heart rate also tended to be smaller (by approximately 35%; *p* = 0.12) following sustained β-adrenergic receptor blockade. The effect of *I*_f_ block by ivabradine on heart rate (figure 6*b*) is a measure of the functional importance of *I*_f_. Block of *I*_f_ by ivabradine had a substantial effect on heart rate at ZT 12 in the control mice, but a significantly smaller effect at ZT 0 in the control mice (figure 6*b*)—this is consistent with a greater functional importance of *I*_f_ at ZT12 (presumably due to a higher expression of HCN channels as a result of the day–night rhythm in *Hcn* transcripts shown in figure 4), which in turn can explain the higher heart rate at ZT12 (figure 1). However, after sustained β-adrenergic receptor blockade, the effect of ivabradine was not statistically different at ZT 0 and ZT 12 (figure 6*b*). This is consistent with the hypothesis that the day–night rhythm in heart rate involves a day–night rhythm in *Hcn4* (and possibly other *Hcn* transcripts), and the reduction in the day–night rhythm in heart rate following sustained β-adrenergic receptor blockade shown in figure 1 is the result of the loss of the day–night rhythm in *Hcn* transcripts. Figure 6*c* shows that in the isolated sinus node after the application of 2 mM Cs^+^ to block *I*_f_ there is no day–night rhythm in the intrinsic pacemaker activity of the sinus node in either control mice or mice following sustained β-adrenergic receptor blockade.

## 3. Discussion

This study demonstrates for the first time that sustained pharmacological block of the sympathetic nervous system diminishes or abolishes the normal day–night rhythm in several sinus node pacemaker ion channel transcripts (figure 4), intrinsic sinus node pacemaking (figure 1*f*,*g*) and the heart rate *in vivo* (figure 1*a*–*e*). This could not be attributed to disruption of the local circadian clock (figure 3), and we propose that rhythmic β-adrenergic input to the heart is necessary to orchestrate day–night rhythms in ion channel expression and intrinsic pacemaker function. The observed dysregulation of tissue-specific transcription factors following sustained β-adrenergic receptor blockade (figure 5) provides a potential mechanism by which this is mediated.

### (a) Mechanism underlying the day–night rhythm in heart rate

Previously we have suggested that a day–night rhythm in the expression of sinus node pacemaker ion channels, in particular HCN4, is either responsible for or plays an important role in the day–night rhythm in heart rate [17]. The results from this study are consistent with this: sustained β-adrenergic receptor blockade diminished or abolished the day–night rhythm in important sinus node pacemaker ion channel transcripts including *Hcn4* (figure 4), the day–night rhythm in the sensitivity of heart rate to block of HCN4 (and other HCN channels) by ivabradine (figure 6*b*), and of course the intrinsic pacemaker activity of the sinus node and the heart rate *in vivo* (figure 1).

### (b) Working hypothesis of how the sympathetic nervous system is involved in the day–night rhythm in heart rate

How is the sympathetic nervous system involved in the day–night rhythm in heart rate? We and others have shown that short-term β-adrenergic receptor blockade has little effect on the day–night rhythm in heart rate [1,17], ruling out a dominant role for short-term regulation of pacemaker single-ion channel conductances. Another possibility is that the sympathetic nervous system controls the local circadian clock and thereby pacemaker ion channel gene transcription; there is some evidence that the sympathetic nervous system can influence the clock [16,28,29]. However, this study showed little or no evidence of this—the day–night rhythms of key circadian clock transcripts were largely unchanged following sustained β-adrenergic receptor blockade (figure 3; see also electronic supplementary material, figure S4). Similar results have been obtained by others: rhythmicity in cardiac clock gene expression is maintained in Dbh^−/−^ mice, which are deficient in *dopamine β-hydroxylase*, an enzyme required for catecholamine synthesis [30]. Furthermore, in studies of sustained blockade of the sympathetic nervous system in mice and rats, rhythmicity in atrial and ventricular clock transcripts continued [20,31].

Our working hypothesis is that the day–night rhythm in sympathetic activity directly causes (or contributes to—see below) a day–night rhythm in ion channel transcription; this explains why the rhythms in ion channel transcription are disrupted with sustained β-adrenergic receptor blockade. The regulation of gene transcription by β-adrenergic signalling can occur via multiple signalling cascades, including those involving the protein kinases Ca^2+^/calmodulin-dependent protein kinase (CaMK) and mitogen-activated protein kinases. Each of these signalling pathways can phosphorylate the ubiquitous transcription factor CREB, which promotes gene expression by binding to cAMP response elements (CREs) at target genes. Transcriptional regulation via these mechanisms has been reported for the L-type Ca^2+^ channel subunits *Cacna2d1* (Ca_v_α2δ) and *Cacnb3* (Ca_v_β3) [32], and various K^+^ channels, *Kcna4* (K_v_1.4), *Kcna5* (K_v_1.5), *Kcnd2* (K_v_4.2) and *Kcnd3* (K_v_4.3) [33,34]. This has not been investigated for *Hcn4*, but conserved CRE motifs are enriched in its promoter region (data not shown). It may be relevant that various CREB subunit transcripts (*Creb1*, *Creb3*, *Creb5*, *Creb3l1* and *Creb3l3*) show a significant day–night rhythm in the mouse sinus node (analysis of data in Wang *et al*. [18]). The day–night rhythm in *Creb1* is shown in electronic supplementary material, figure S7. β-Adrenergic signalling-mediated regulation of pacemaker ion channel transcription may be via transcription factors known to regulate pacemaking ion channel expression. As shown in figure 5, sustained β-adrenergic receptor blockade resulted in the loss of the day–night rhythm in transcripts for (i) the sinus node-specific *Shox2* [35], (ii) *Tbx3* and *Tbx18*, which regulate the pacemaking phenotype and can promote ectopic *Hcn4* expression [36,37], and (iii) *Mef2c*, which is a direct regulator of *Hcn4* [38]. β-Adrenergic regulation of MEF2 transcription factors at least is well-established [38].

Although sustained β-adrenergic receptor blockade results in the loss of the day–night rhythm in ion channel transcripts, including *Hcn4*, in the sinus node (figure 4), we have previously shown that there is a functioning circadian clock in the sinus node, and cardiac-specific knockout of *Bmal1*, a key clock transcript, also results in the loss of the day–night rhythm in *Hcn4* [17]. There is a well-established day–night rhythm in plasma cortisol, and suppression of this results in the loss of the day–night rhythm in ion channel transcripts in the left ventricle (unpublished findings). Finally, in mouse heart, knockout of *Klf15* has been reported to result in the loss of the day–night rhythm of 75% of the normally rhythmic transcripts [39]. These seemingly conflicting findings can be explained by combinatorial control of gene expression—because the number of genes far exceeds the number of transcription factors, many genes are controlled by several different transcription factors (binding at a *cis*-regulatory or enhancer site), with a specific combination needed for the gene to be transcribed [40]. For example, it is feasible that transcription of *Hcn4* requires both adrenergic signalling (and CREB) and the local clock (and BMAL1), which may explain why abrogation of either leads to the loss of the day–night rhythm in *Hcn4*.

## 4. Conclusion

This study has shown an important but non-canonical role of the sympathetic nervous system in the day–night rhythm in heart rate. Surprisingly, this does not involve the short-term regulation of pacemaker ion channels. Instead, it involves the regulation of rhythmic pacemaker ion channel transcription.

## 5. Material and methods

All animal experiments were performed on male C57BL/6J mice approximately 10 weeks of age and were approved by the University of Manchester in accordance with the UK Animals (Scientific Procedures) Act 1986. Propranolol was administered to some mice via the drinking water as in previous studies (e.g. [21]); control mice received standard drinking water (propranolol-free). The heart or beating rate was recorded from the ECG in conscious freely moving mice using telemetry, in conscious but constrained mice using an ECGenie, in anaesthetized mice using standard electrodes, and in the isolated sinus node using extracellular potential recording. Finally, mice were sacrificed by cervical dislocation and biopsies collected from the sinus node and mid-left ventricular free wall. RNA was isolated and reverse transcribed to generate cDNA and then the qPCR was used to measure the expression of 90 transcripts. Statistical analyses were carried out using a range of tests. Means ± s.e.m. are shown in figures. Full details of the methods used are given in the electronic supplementary material.

## Data Availability

The data are provided in the electronic supplementary material [41].

## References

[1] Black N, D'Souza A, Wang Y, Piggins H, Dobrzynski H, Morris G, Boyett MR. 2018 Circadian rhythm of cardiac electrophysiology, arrhythmogenesis, and the underlying mechanisms. Heart Rhythm **16**, 298-307. (10.1016/j.hrthm.2018.08.026) 30170229 PMC6520649

[2] Sutherland GA. 1929 The pulse rate and range in health and disease during childhood. Q. J. Med. **22**, 519-529.

[3] Boas EP, Weiss MM. 1929 The heart rate during sleep as determined by the cardiotachometer: its clinical significance. J. Am. Med. Assoc. **92**, 2162-2168. (10.1001/jama.1929.02700520014006)

[4] Zhang H, Butters T, Adeniran I, Higham J, Holden AV, Boyett MR, Hancox JC. 2012 Modeling the chronotropic effect of isoprenaline on rabbit sinoatrial node. Front. Physiol. **3**, 241. (10.3389/fphys.2012.00241) 23060799 PMC3459472

[5] Wickman K, Krapivinsky G, Corey S, Kennedy M, Nemec JAN, Medina I, Clapham DE. 1999 Structure, G protein activation, and functional relevance of the cardiac G protein-gated K^+^ channel, *I*_KACh_. Ann. NY Acad. Sci. **868**, 386-398. (10.1111/j.1749-6632.1999.tb11300.x) 10414308

[6] Porro A, Thiel G, Moroni A, Saponaro A. 2020 Cyclic AMP regulation and its command in the pacemaker channel HCN4. Front. Physiol. **11**, 771. (10.3389/fphys.2020.00771) 32733276 PMC7358946

[7] Trautwein W, Hescheler J. 1990 Regulation of cardiac L-type calcium current by phosphorylation and G proteins. Annu. Rev. Physiol. **52**, 257-274. (10.1146/annurev.ph.52.030190.001353) 2158764

[8] Vandewalle G, Middleton B, Rajaratnam SM, Stone BM, Thorleifsdottir B, Arendt J, Dijk DJ. 2007 Robust circadian rhythm in heart rate and its variability: influence of exogenous melatonin and photoperiod. J. Sleep Res. **16**, 148-155. (10.1111/j.1365-2869.2007.00581.x) 17542944

[9] Monfredi O et al. 2014 Biophysical characterisation of the under-appreciated and important relationship between heart rate variability and heart rate. Hypertension **64**, 1334-1343. (10.1161/HYPERTENSIONAHA.114.03782) 25225208 PMC4326239

[10] Ogawa M et al. 2007 Left stellate ganglion and vagal nerve activity and cardiac arrhythmias in ambulatory dogs with pacing-induced congestive heart failure. J. Am. Coll. Cardiol. **50**, 335-343. (10.1016/j.jacc.2007.03.045) 17659201

[11] Bussey CT. 2019 Direct recordings of cardiac sympathetic and vagal parasympathetic nerve activity to clarify the underlying mechanisms of circadian heart rhythm. Proc. Physiol. Soc. **43**, SA034.

[12] Jung BC et al. 2006 Circadian variations of stellate ganglion nerve activity in ambulatory dogs. Heart Rhythm **3**, 78-85. (10.1016/j.hrthm.2005.09.016) 16399059

[13] Turton MB, Deegan T. 1974 Circadian variations of plasma catecholamine, cortisol and immunoreactive insulin concentrations in supine subjects. Clin. Chim. Acta **55**, 389-397. (10.1016/0009-8981(74)90014-x) 4412449

[14] De Boer SF, Van Der Gugten J. 1987 Daily variations in plasma noradrenaline, adrenaline and corticosterone concentrations in rats. Physiol. Behav. **40**, 323-328. (10.1016/0031-9384(87)90054-0) 3659148

[15] Scheer FAJL, Hu K, Evoniuk H, Kelly EE, Malhotra A, Hilton MF, Shea SA. 2010 Impact of the human circadian system, exercise, and their interaction on cardiovascular function. Proc. Natl Acad. Sci. USA **107**, 20 541-20 546. (10.1073/pnas.1006749107) PMC2996667 21059915

[16] Jesus ICG et al. 2021 Molecular basis of Period 1 regulation by adrenergic signaling in the heart. FASEB J. **35**, e21886. (10.1096/fj.202100441R) 34473369

[17] D'Souza A et al. 2021 A circadian clock in the sinus node mediates day-night rhythms in *Hcn4* and heart rate. Heart Rhythm **18**, 801-810. (10.1016/j.hrthm.2020.11.026) 33278629 PMC8073545

[18] Wang Y, Anderson C, Dobrzynski H, Hart G, D'Souza A, Boyett MR. 2021 RNAseq shows an all-pervasive circadian rhythm in the transcriptome of the pacemaker of the heart, the sinus node. Scient. Rep. **11**, 3565. (10.1038/s41598-021-82202-7) PMC7878777 33574422

[19] Witte K, Engelhardt S, Janssen BJ, Lohse M, Lemmer B. 2004 Circadian and short-term regulation of blood pressure and heart rate in transgenic mice with cardiac overexpression of the β1-adrenoceptor. Chronobiol. Int. **21**, 205-216. (10.1081/cbi-120037801) 15332342

[20] Tong M, Watanabe E, Yamamoto N, Nagahata-Ishiguro M, Maemura K, Takeda N, Nagai R, Ozaki Y. 2013 Circadian expressions of cardiac ion channel genes in mouse might be associated with the central clock in the SCN but not the peripheral clock in the heart. Biol. Rhythm Res. **44**, 519-530. (10.1080/09291016.2012.704801) 23420534 PMC3570950

[21] Fabritz L et al. 2010 Autonomic modulation and antiarrhythmic therapy in a model of long QT syndrome type 3. Cardiovasc. Res. **87**, 60-72. (10.1093/cvr/cvq029) 20110334 PMC2883895

[22] Gao B, Cutler MG. 1992 Effects of acute and subchronic administration of propranolol on the social behaviour of mice; An ethopharmacological study. Neuropharmacology **31**, 749-756. (10.1016/0028-3908(92)90036-O) 1528404

[23] Hughes ME, Hogenesch JB, Kornacker K. 2010 JTK_CYCLE: an efficient nonparametric algorithm for detecting rhythmic components in genome-scale data sets. J. Biol. Rhythms **25**, 372-380. (10.1177/0748730410379711) 20876817 PMC3119870

[24] Linscheid N et al. 2019 Quantitative proteomics and single-nucleus transcriptomics of the sinus node elucidates the foundation of cardiac pacemaking. Nat. Commun. **10**, 2889. (10.1038/s41467-019-10709-9) 31253831 PMC6599035

[25] Liang D et al. 2021 Cellular and molecular landscape of mammalian sinoatrial node revealed by single-cell RNA sequencing. Nat. Commun. **12**, 287. (10.1038/s41467-020-20448-x) 33436583 PMC7804277

[26] Schroder EA et al. 2015 The cardiomyocyte molecular clock regulates the circadian expression of *Kcnh2* and contributes to ventricular repolarization. Heart Rhythm **12**, 1306-1314. (10.1016/j.hrthm.2015.02.019) 25701773 PMC4541807

[27] Li L, Li H, Tien CL, Jain MK, Zhang L. 2020 Kruppel-like factor 15 regulates the circadian susceptibility to ischemia reperfusion injury in the heart. Circulation **141**, 1427-1429. (10.1161/CIRCULATIONAHA.119.041664) 32339045 PMC7197441

[28] Durgan DJ et al. 2005 The intrinsic circadian clock within the cardiomyocyte. Am. J. Physiol. Heart Circ. Physiol. **289**, H1530-H1541. (10.1152/ajpheart.00406.2005) 15937094

[29] Beesley S, Noguchi T, Welsh DK. 2016 Cardiomyocyte circadian oscillations are cell-autonomous, amplified by β-adrenergic signaling, and synchronized in cardiac ventricle tissue. PLoS ONE **11**, e0159618. (10.1371/journal.pone.0159618) 27459195 PMC4961434

[30] Reilly DF et al. 2008 Peripheral circadian clock rhythmicity is retained in the absence of adrenergic signaling. Arterioscler. Thromb. Vasc. Biol. **28**, 121-126. (10.1161/atvbaha.107.152538) 17975121 PMC2752700

[31] Ushijima K, Maekawa T, Ishikawa-Kobayashi E, Ando H, Shiga T, Fujimura A. 2013 Influence of beta-blockers on the myocardial mRNA expressions of circadian clock- and metabolism-related genes. J. Am. Soc. Hypertension **7**, 107-117. (10.1016/j.jash.2012.12.007) 23394803

[32] Al Katat A, Zhao J, Calderone A, Parent L. 2022 Sympathetic stimulation upregulates the Ca^2+^ channel subunit, Ca_V_α2*δ*1, via the β1 and ERK 1/2 pathway in neonatal ventricular cardiomyocytes. Cells **11**, 188. (10.3390/cells11020188) 35053304 PMC8774121

[33] Bru-Mercier G, Deroubaix E, Capuano V, Ruchon Y, Rücker-Martin C, Coulombe A, Renaud JF. 2003 Expression of heart K^+^ channels in adrenalectomized and catecholamine-depleted reserpine-treated rats. J. Mol. Cell. Cardiol. **35**, 153-163. (10.1016/s0022-2828(02)00290-0) 12606256

[34] Mori Y, Matsubara H, Folco E, Siegel A, Koren G. 1993 The transcription of a mammalian voltage-gated potassium channel is regulated by cAMP in a cell-specific manner. J. Biol. Chem. **268**, 26 482-26 493. (10.1016/S0021-9258(19)74340-3) 8253777

[35] van Eif V et al. 2019 Transcriptome analysis of mouse and human sinoatrial node cells reveals a conserved genetic program. Development **146**, dev173161. (10.1242/dev.173161) 30936179

[36] Hoogaars WMH et al. 2007 Tbx3 controls the sinoatrial node gene program and imposes pacemaker function on the atria. Genes Dev. **21**, 1098-1112. (10.1101/gad.416007) 17473172 PMC1855235

[37] Kapoor N, Liang W, Marban E, Cho HC. 2013 Direct conversion of quiescent cardiomyocytes to pacemaker cells by expression of *Tbx18*. Nat. Biotechnol. **31**, 54-62. (10.1038/nbt.2465) 23242162 PMC3775583

[38] Kuratomi S et al. 2009 The cardiac pacemaker-specific channel *Hcn4* is a direct transcriptional target of MEF2. Cardiovasc. Res. **83**, 682-687. (10.1093/cvr/cvp171) 19477969

[39] Zhang L, Prosdocimo DA, Bai X, Fu C, Zhang R, Campbell F, Liao X, Coller J, Jain MK. 2015 KLF15 establishes the landscape of diurnal expression in the heart. Cell Rep. **13**, 2368-2375. (10.1016/j.celrep.2015.11.038) 26686628

[40] Reményi A, Schöler HR, Wilmanns M. 2004 Combinatorial control of gene expression. Nat. Struct. Mol. Biol. **11**, 812-815. (10.1038/nsmb820) 15332082

[41] Anderson C, Forte G, Hu W, Zhang H, Boyett MR, D'Souza A. 2023 Non-canonical role of the sympathetic nervous system in the day–night rhythm in heart rate. Figshare. (10.6084/m9.figshare.c.6492838) PMC10150211 37122216

